# Social cohesion and artistic resources, Two Clinical cases.

**DOI:** 10.1192/j.eurpsy.2023.1848

**Published:** 2023-07-19

**Authors:** N. Simone

**Affiliations:** DSM Ancona, ASUR MARCHE, Ancona, Italy

## Abstract

**Introduction:**

Two emblematic clinical cases will be addressed in this poster. The purpose of the poster is to relate the aspect of how the artistic resources of psychiatric patients are useful to improve social cohesion.

**Objectives:**

Correlate psychotherapy work on superior defenses to social cohesion and clinical improvement.

**Methods:**

Psychopharmacotherapy. Psychoanalytically oriented psychotherapy. Psychiatric rehabilitation.

Social jutice idex (Schraad-Tishler, 2015) and BES (Istat 2015) as sources of social cohesion indicators.

**Results:**

In the first case we have a man with ciclotimic personality disorder and substace abuse with psicotic episodes. Previously, the patient underwent repeated hospitalizations . Once the therapeutic relationship was hooked up and introduced in addition to pharmacological treatment, a psychotherapy also aimed at activating personal resources, the patient began to take an interest in the activity of a street artist. Over time and with the improvement of this activity, aspects of social cohesion have been highlighted in the social context. Social relations both linked to work and affective activity, as well as friends; improvement of the economic situation both through the economic return of this activity and through associative loans linked to this activity; an improvement of social inclusion and non-discrimination in the cities where the artistic activities were carried out; the use of fixed environmental spaces where the activity can be carried out. These aspects have favored a recovery that has reached the complete economic independence of the patient, the establishment of a family unit of their own, the progressive remission of the toxicophilic relapses and therefore the psychotic imbalances they induce.

In a second case, we have a 30-year-old patient with a severe depressive disorder, with narcissistic, paranoid and obsessive personality traits, graduated from the Academy of Fine Arts and with an artistic production both before and after the illness. In addition pharmacological therapy, the patient began psychotherapeutic interventions on the enhancement of superior defenses such as sublimation through artistic activity. So she began to rededicate herself to the artistic activity , she however progressively improved the recovery aspects for a long period of time up to living, working and emotional autonomy. During this path the aspects of social cohesion were also decisive, such an economic autonomy linked to the work activity of an employee in an art gallery, a progressive social inclusion, the conquest of spaces where you can exhibit your works and an increase in trust both towards the family and towards health workers and towards friends and work relationships.

**Image:**

**Figure:**
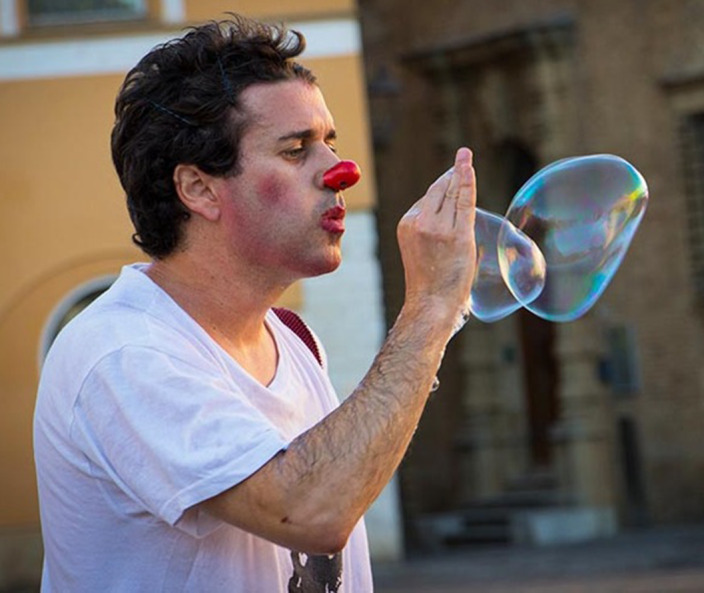

**Image 2:**

**Figure:**
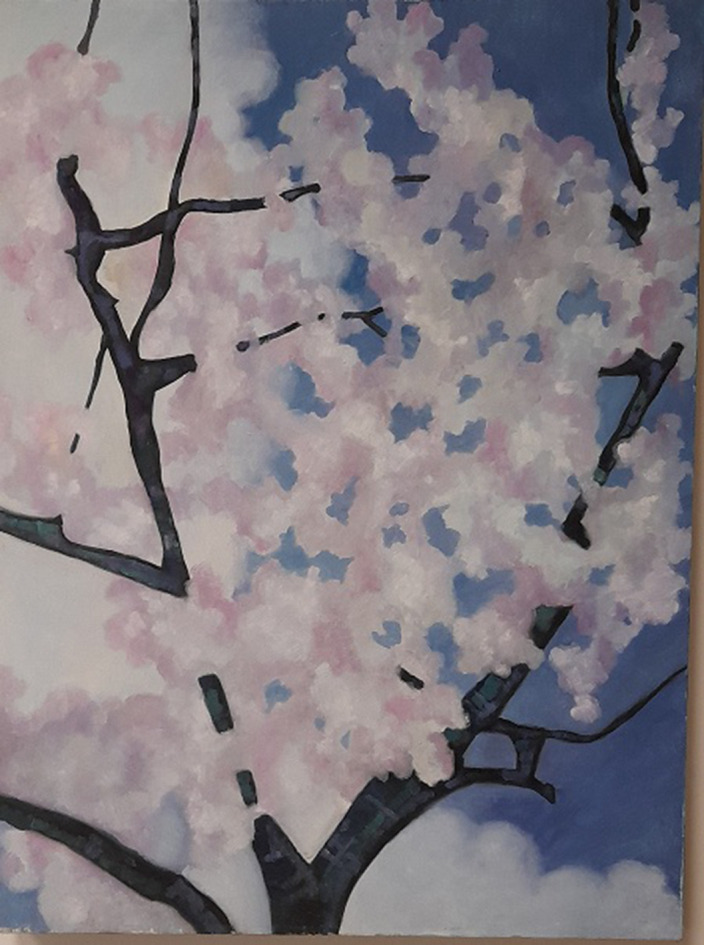

**Image 3:**

**Figure:**
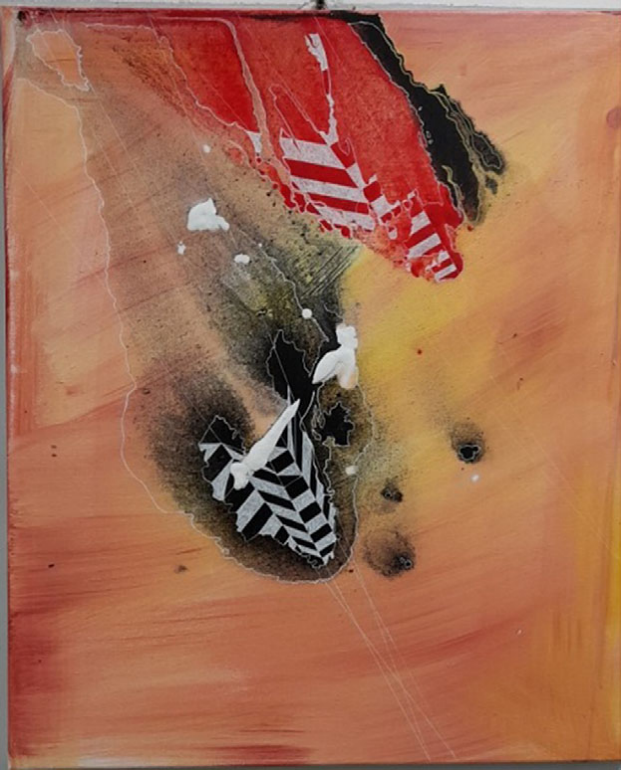

**Conclusions:**

In this Clinical cases and others not reported exist correlation between psychotherapy work on superior defenses to social cohesion and clinical improvement.

**Disclosure of Interest:**

None Declared

